# Differential DNA methylation correlates with response to methotrexate in rheumatoid arthritis

**DOI:** 10.1093/rheumatology/kez411

**Published:** 2019-10-10

**Authors:** Nisha Nair, Darren Plant, Suzanne M Verstappen, John D Isaacs, Ann W Morgan, Kimme L Hyrich, Anne Barton, Anthony G Wilson, the MATURA investigators

**Affiliations:** 1 Centre of Genetics & Genomics Versus Arthritis, Manchester Academic Health Sciences Centre, The University of Manchester; 2 NIHR Manchester BRC, Manchester University Foundation Trust; 3 Centre for Epidemiology Versus Arthritis, Manchester Academic Health Sciences Centre, The University of Manchester, Manchester; 4 Institute of Cellular Medicine, Newcastle University, Newcastle upon Tyne UK; and NIHR Newcastle BRC, Newcastle upon Tyne Hospitals NHS Foundation Trust, Newcastle upon Tyne; 5 Leeds Institute of Rheumatic and Musculoskeletal Medicine, School of Medicine, University of Leeds, Leeds, UK and NIHR Leeds BRC, Leeds Teaching Hospitals NHS Trust, Leeds, UK; 6 University College Dublin School of Medicine and Medical Science, University College Dublin, Dublin, Ireland

**Keywords:** rheumatoid arthritis, treatment response, methotrexate, pharmacogenetics, DNA methylation, epigenetics, DMARD

## Abstract

**Objectives:**

Identifying blood-based biomarkers that predict treatment response in RA is a clinical priority. We investigated differential DNA methylation as a candidate biomarker of response for the first-line drug used in RA, MTX.

**Methods:**

DNA methylation was measured in DNA samples from individuals recruited to the Rheumatoid Arthritis Medication Study. Differentially methylated positions were compared between whole blood samples collected at baseline and at 4 weeks from patients who, by 6 months, had a good (*n* = 34) or poor response (*n* = 34) to MTX using linear modelling, adjusting for gender, age, cell composition, baseline 28-joint disease activity score (DAS28) and smoking status. Analyses also compared methylation with changes in DAS28 and changes in swollen joint count and tender joint count, and changes in CRP over the initial 6 months after MTX commencement. Differentially methylated positions showing significant differences with any response parameter were tested using pyrosequencing in an independent group of 100 patients from the Rheumatoid Arthritis Medication Study.

**Results:**

In the discovery group, two CpG sites showed methylation changes at 4 weeks associated with clinical EULAR response by 6 months. Significant changes in methylation for three differentially methylated positions associated with change in tender joint counts, three with change in swollen joint count and a further four with change in CRP. Of the 12 CpGs, four showed replicated association in an independent dataset of samples from the Rheumatoid Arthritis Medication Study.

**Conclusion:**

These data represent an advance on current practice by contributing to a personalized medicine strategy allowing an escalation or change in therapy as early as 4 weeks.

**Rheumatology key messages**

- Up to 40% of rheumatoid arthritis patients do not respond adequately to methotrexate treatment.
- Four CpGs measured after 4 weeks of treatment correlated with improvement in disease activity.
- Integrating CpGs with other early biomarkers may predict improvement in disease activity at 6 months.

## Introduction

Multiple studies have shown that patients with RA who receive prompt and efficacious treatment resulting in sustained remission are less likely to experience long-term disability and premature mortality [1–3]. The first choice conventional synthetic disease-modifying antirheumatic drug (csDMARD) treatment for RA is MTX; however, up to 40% of patients do not respond adequately to treatment after 2 years [4]. A clinical decision regarding response to MTX is generally made at 6 months following treatment initiation. During that time, prolonged inflammation can cause lasting disability in patients who are non-responsive to treatment. Therefore, it is imperative to identify earlier biomarkers of response so that patients can be placed on a more effective personalized treatment regimen earlier in their disease course.

One possible biomarker of MTX response is DNA methylation, in which a methyl group is added to a cytosine–guanine (C–G) dinucleotide (CpG). Approximately 70–80% of all CpGs across the genome are methylated [5] and typically cluster in gene promoter regions, known as CpG islands, which are usually hypomethylated in transcriptionally active genes [6]. There is increasing evidence for a role of DNA methylation in susceptibility to RA. For example, the largest epigenome-wide association study (EWAS) for RA susceptibility to date compared DNA methylation at 485 000 CpG sites in 354 RA cases and 337 healthy controls and reported 10 differentially methylated CpG sites within the MHC region at known RA genetic risk loci [7]. The authors suggested that some genetic associations at the MHC region may be mediated by DNA methylation by affecting gene regulation or alternative splicing [7].

DNA methylation is also a candidate biomarker for treatment response. For example, one study compared DNA methylation signatures in whole blood from 36 good and 36 poor responders to etanercept, and two differentially methylated positions (DMPs) mapping to the *LRPAP1* gene were identified [8]. Methylation levels of these DMPs were also associated with genotype of the rs3468 variant in *LRPAP1* and the genetic association with response was replicated in an independent set of anti-TNF-treated subjects (*n* = 1204). Another study investigated DNA methylation in CD4^+^ T cells from JIA patients; 145 DMPs were identified between cases and controls (false discovery rate adjusted *P* < 0.1), but just 11 DMPs remained associated when four MTX-treated individuals were removed, suggesting a possible role of MTX on DNA methylation in CD4^+^ T cells in JIA cases [9]. However, no previous studies have investigated DNA methylation as a biomarker of future response to MTX.

We, therefore, performed an EWAS to identify DMPs between good and poor responders at 6 months after initiating MTX treatment for the first time in patients with RA, both at baseline and following 4 weeks of therapy, in order to identify a very early biomarker of response. Using genome-wide genetic data, we further investigated the correlation of differential methylation with genotype to identify whether methylation quantitative trait loci (meQTLs) were present and could potentially act as a surrogate marker.

## Methods

### Patient selection

The Rheumatoid Arthritis Medication Study (RAMS) is a UK multicentre 1 year observational study of patients diagnosed with RA or inflammatory polyarthritis who commence MTX for the first time. Whole blood samples were collected pre-treatment (baseline) and 4 weeks following therapy. Sera were extracted from the whole blood samples and measured for CRP levels. DNA was extracted from whole blood samples collected at baseline and from samples collected following 4 weeks of MTX therapy.

Clinical information was collected at baseline and 6 months thereafter, including 28 swollen joint count (SJC) and tender joint count (TJC). Using data for CRP, SJC, TJC and GH (patient global health; patient reported global health assessment ranging from 0 mm = best and 100 mm = worst), the patients’ 28-joint disease activity score (DAS28) was calculated at baseline and 6 months. Response to MTX was determined at 6 months using the EULAR criteria for response where good response was defined as having DAS28 ⩽3.2 and >1.2 improvement of DAS28 at 6 months, and poor response was defined as having DAS28 >5.1 and ⩽0.6 improvement of DAS28 at 6 months [10]. Patients who had improved the most by 6 months were selected as good responders, and patients whose DAS28 showed the least improvement (or worsened) by 6 months were selected as poor responders for the genome-wide methylation analysis. Baseline characteristics of the patients selected for the genome-wide methylation analyses are found in Table 1. RAMS was approved by the Central Manchester NHS Research Ethics Committee (ref. 08/H1008/25) and all patients provided written consent.

**Table 1:** Baseline patient characteristics in the RAMS cohort

	Good responders (*n* = 34)	Poor responders (*n* = 34)
Female, *n* (%)	26 (76.5)	28 (82.3)
Age at baseline, mean (s.d.), years	58.8 (14.5)	58.3 (14.2)
Baseline DAS28, mean (s.d.)	4.9 (1.0)	4.3 (1.4)
Swollen joint count, median (IQR)	6.5 (1–20)	4.5 (0–18)
Tender joint count, median (IQR)	9.5 (4–25)	9 (0–27)
CRP, median (IQR), mg/µl	7.5 (1–76)	3.5 (0–28)
Smoking status at baseline, *n* (%)		
Never smoked	17 (50)	15 (44.)
Current smoker	6 (17.6)	9 (26.4)
Ex-smoker	11 (32.4)	10 (29.4)

Clinical and demographic data were collected at baseline (pre-treatment) and at 6 months following treatment. Results are presented as mean (s.d.) or median (IQR) if data are skewed. IQR: interquartile range.

### Epigenome-wide methylation analysis

Five hundred nanograms of DNA was bisulphite converted using an optimized protocol of the manufacturer (Zymo Research, Irvine, CA, USA). Epigenome-wide methylation was measured using HumanMethylation450 BeadChips according to the manufacturer’s protocol (Illumina, San Diego, CA, USA).

All statistical analyses were performed in R 3.2.0 (R Foundation for Statistical Computing, Vienna, Austria). Using previously validated methodology, linear models created using the *limma* package were used to test for association between CpG methylation and treatment response in pre-treatment samples and in those taken at 4 weeks, as well as differential methylation between the two time points in both the good and the poor responder groups, separately [8, 11]. The changes in DAS28 score and its components (SJC, TJC, CRP) were compared with changes in methylation at baseline and 4 weeks separately, and with changes between baseline and 4 weeks for each responder group. By looking for these changes in the individual responder groups improved the power to detect smaller changes in methylation, which associate with the DAS28 and its components in a more extreme phenotype The GH component of the DAS was not included in these analyses as it is a very subjective variable and does not always correlate with other objective measures of RA disease activity [12]. The baseline DAS28 score, sex and age were included as covariates in the model as well as cell type composition using the validated Houseman method [13], and smoking status (never smoked, past smoker, current smoker) since whole blood is a heterogeneous group of cell types with each cell type having its own distinct methylation profile, and also smoking is also known to influence global DNA methylation [13–16]. *P*-values were adjusted using the Benjamini and Hochberg method to control the false discovery rate [11]. Power to detect differential DNA methylation between responder groups was estimated using established analytical techniques, and DMPs with an unadjusted *P*-value <1 × 10^−6^ (arbitrary threshold) was deemed as a suggestive association and prioritized for replication [17].

### Replication using pyrosequencing

An independent replication cohort for pyrosequencing was selected from RAMS. This cohort included 28 extremely good and 35 extremely poor responders using the same EULAR definitions as in the discovery cohort, as well 36 intermediate responders. Intermediate response was determined using the EULAR criteria where patients had an improvement of DAS28 at 6 months >0.6 and ⩽1.2 with a present DAS28 ⩽3.2 or >3.2 and ⩽5.1, or an improvement at 6 months by >1.2 with present DAS28 >3.2 and ⩽5.1, or >5.1 [10]. All patients had DNA samples taken at baseline (pre-treatment) and following 4 weeks of treatment with MTX.

Pyrosequencing assays were designed or selected to target DMPs with evidence for association from the array-based genome-wide analysis (Qiagen, Hilden, Germany). For each assay, 10 ng of bisulphite-converted DNA was amplified by PCR as suggested by the manufacturer (Qiagen). The PCR product (10–20 μl) was bound to streptavidin–Sepharose High Performance Beads (GE Healthcare, Chalfont St. Giles, UK), and washed with 70% ethanol, PyroMark denaturation solution, and PyroMark wash buffer (Qiagen) before being added to 0.3 μM sequencing primer diluted to 25 μl in annealing buffer (Qiagen). The solution was heated to 80°C for 2 min to allow the biotin-labelled DNA strand to anneal to the primers, and then cooled to room temperature before being loaded into the pyrosequencer, PyroMark Q24 (Qiagen). The percentage methylation value at each CpG site was compared with the β-values for that site derived from the epigenome wide analysis. Statistical analysis using a linear regression model for comparisons between time point, response status and change in DAS28 component at each time point and within responder groups for baseline and 4 weeks samples separately were repeated to determine whether an association was replicated in the same direction and was statistically significant (*P*-value <0.05).

### Pathway analysis

To gain a better understanding of the biological function of these replicated methylation changes, pathway analysis was performed using the epigenome-wide data. For any CpG site that was replicated in the pyrosequencing analyses, the top 1000 CpGs in the epigenome-wide results for its specific comparison (e.g. change in SJC with baseline samples) were investigated for potential biological pathways inferred by Gene Ontology using the *missMethyl* package in R [18]. A *P*-value cut-off <10^−5^ was used to determine statistical significance of the biological pathways identified.

### meQTL analysis

The DNA samples from the patients in this study were also genotyped using the Illumina HumanCoreExome-24-v1-0 according to the manufacturer’s protocol (Illumina). The raw genotype data were assessed using GenomeStudio, where samples <90% call rate were excluded and the data was clustered. Data were exported to PLINK v1.07 [19] where variants and individuals with <98% call rate were excluded. Each chromosome was aligned to The 1000 Genomes Project Phase 3 reference panel using shapeit.v2.r837 [20]. The imputation was performed using impute2_2.3.2. From the imputed dataset, whole chromosomal regions pertaining to the replicated CpG regions were extracted using PLINK v1.07. Using the methylation values from the epigenome-wide data and the corresponding genome-wide single nucleotide polymorphism (SNP) data, SNPs mapped to within 1 million bases around the replicated CpGs were investigated for *cis*-acting meQTLs using the R package, *matrixEQTL* [21].

## Results

### Cohort characteristics

Baseline characteristics of the 34 good responders and 34 poor responders to MTX tested in the genome-wide array-based study are shown in Table 1. No significant differences were observed between treatment response groups except for baseline DAS28 score, which was higher in the good responder group compared with the poor responder group, and was subsequently added as a covariate in all analyses. This study had 80% power to detect a mean methylation difference of 7% between good responders and poor responders at the 5% significance threshold.

### Discovery epigenome-wide association results

A total of 422 557 probes were available for further analyses in 34 poor responders and 34 good responders at both time points, where response was determined using the EULAR criteria at 6 months. The linear regression in *limma* identified two DMPs in DNA samples taken at the 4-week time point, which differed between good and poor responders (*P* < 1 × 10^−6^). There was no differential methylation observed between good and poor responders in the baseline DNA samples, or between time points in either the good responder group or the poor responder group. Correlations between methylation changes between baseline and 4 weeks and changes in the individual DAS28 components were also compared separately in the good and poor responder groups. Subsequently, changes in a further 10 DMPs between baseline and 4 weeks were correlated with changes in individual DAS components either at the two specific time points or in the separate responder groups where the change was seen between baseline and 4 weeks (*P* < 1 × 10^−6^): three DMPs were associated with change in TJC, three other DMPs were associated with change in SJC, and a further four DMPs were associated with change in CRP (Tables 2 and 3).

**Table 2:** Two DMPs associated with treatment response after 4 weeks of MTX treatment

CpG site	Average methylation in GR, %	Average methylation in PR, %	*P*-value	Nearest gene	Relation to gene
cg21040096	77.3	87.3	2.79 × 10^−7^	*RPH3AL*	5′UTR
cg09894276	87.7	80.4	3.62 × 10^−7^	*WDR27*	Gene intron

Comparison of DNA methylation in samples taken after 4 weeks of treatment identified two differentially methylated positions (DMPs) (*P*-value < 10^−6^) that showed differences in methylation between good (GR) and poor (PR) responders. Response status was based on EULAR response criteria using the composite DAS28 score calculated after 6 months of treatment. The CpG sites were situated in the 5′-untranslated region (5′UTR) and intron of their respective nearest genes.

**Table 3:** Top 10 DMPs after 4 weeks of treatment associated with response

CpG site	Parameter	Change in DAS28 component	*P*-value	Nearest gene	Relation to nearest gene
cg13793478	TJC at 4 weeks	+0.07	9.78 × 10^−8^	*FOXD4*	∼
cg19357849	TJC at 4 weeks	+0.05	7.73 × 10^−7^	*ELAVL1*	TSS1500
cg06201514	TJC in good responders	+0.06	2.90 × 10^−7^	*MYT1L*	Gene body
cg23700278	SJC in good responders	−0.13	1.97 × 10^−10^	*ADRA2C*	∼
cg27427581	SJC in good responders	−0.10	6.45 × 10^−9^	*GATA3*	∼
cg16682276	SJC in poor responders	+0.03	1.25 × 10^−6^	*GNMT*	TSS1500
	CRP at baseline	−0.06	5.01 × 10^−8^		
cg04334751	CRP at 4 weeks	−0.16	1.31 × 10^−12^	*MIR182*	∼
	CRP in poor responders	−0.32	2.80 × 10^−14^		
cg08799865	CRP at 4 weeks	−0.19	1.17 × 10^−13^	*NT5C2*	TSS200
cg26764200	CRP in good responders	−0.23	8.71 × 10^−10^	*SNORD84; BAT1*	TSS1500
	CRP at baseline	−0.06	3.77 × 10^−11^		
cg04115006	CRP at 4 weeks	−0.14	1.84 × 10^−11^	*C14orf180*	3′UTR
	CRP at 4 weeks in poor responders	−0.34	3.97 × 10^−20^		

Top 10 CpG sites where every 1% increase in methylation corresponded to change in DAS28 component. Where time point features as a parameter, the changes in methylation and in DAS28 component score at 6 months were found at that time point. Where good or poor responder features as a parameter, the changes in DAS28 component correlated with changes in methylation between time points in that responder group. TSS1500/200: 1500/200 base pairs upstream of the transcription start site; 3′UTR: 3′-untranslated region; ∼: not an annotated region.

### Replication of epigenome-wide findings using pyrosequencing

Significant differential methylation was replicated at four of the 12 CpGs (cg26764200, cg23700278, cg27427581, cg04334751), for the same trait and with the same direction of effect in the independent data set of a further 100 samples from the RAMS study (*P* < 0.01) (Table 4). The results of Table 4 show that small decreases in the SJC or CRP correlated with modest increases in methylation. While these changes seem small, considering, for example, cg23700278, where every 0.20 decrease in SJC correlated with a 0.17% increase in methylation, an improvement of SJC by 5 would correspond to 4.25% increase in methylation at 4 weeks.

**Table 4:** Four CpG associations were replicated in the pyrosequencing dataset

CpG	Δ DAS component	Δ methylation (%)	Time point/response	*P*-value	Gene
cg23700278	−0.20 (SJC)	+0.17	GR	0.003	*ADRA2C*
cg27427581	−0.06 (SJC)	+0.23	GR	0.005	*GATA3*
cg04334751	−0.15 (CRP)	+0.15	BL	0.0002	*MIR182*
cg26764200	−0.12 (CRP)	+0.21	GR	0.005	*SNORD84; BAT1*

Two CpG sites were associated with an improvement of swollen joint count (SJC) at 6 months with increased methylation in good responders (GR) by 4 weeks, and two additional CpG sites were associated with an improvement of CRP levels at 6 months with increased methylation in baseline (BL) samples, and in GR by 4 weeks.

### Pathway analyses of replicated associations

In order to find evidence of involvement of these replicated CpG sites in relevant biological pathways, the top 1000 CpG associations from the epigenome-wide analysis for change in SJC in good responders, change in CRP in baseline samples, and change in CRP in good responders were analysed using the R package, *missMethyl* [18]. Significant interactions in biological pathways (*P*-value <10^−5^) are reported in Tables 5 and 6 alongside *P*-values that were adjusted for false discovery rate. The most strongly associated pathways in both analyses involved cell adhesion. There were no significant pathways reported for change in CRP in good responders.

**Table 5:** Top gene ontology pathways associated with change in SJC in good responders

Gene Ontology term		Overall number of genes in pathway	Number of input genes in pathway	*P*-value	FDR adjusted
GO: 0007156	Homophilic cell adhesion via plasma membrane adhesion molecules	145	26	2.51 × 10^−15^	5.04 × 10^−11^
GO: 0098742	Cell–cell adhesion via plasma membrane adhesion molecules	204	27	1.06 × 10^−13^	1.07 × 10^−9^
GO: 0005509	Calcium ion binding	648	39	2.48 10^−10^	1.66 × 10^−6^
GO: 0031224	Intrinsic component of membrane	4936	111	5.29 × 10^−6^	0.025616
GO: 0007155	Cell adhesion	1350	50	8.17 × 10^−6^	0.025616
GO: 0016021	Integral component of membrane	4823	108	8.51× 10^−6^	0.025616
GO: 0022610	Biological adhesion	1355	50	8.92 × 10^−6^	0.025616
GO: 0098609	Cell–cell adhesion	819	34	3.17 × 10^−5^	0.079549
GO: 0007268	Synaptic transmission	687	31	6.60 × 10^−5^	0.147292
GO: 0044425	Membrane part	5946	125	1.12 × 10^−4^	0.22557

The overall number of genes relates to the total number of genes in the pathway as annotated by Gene Ontology. The number of input genes in the pathway relates to the genes captured by the CpG sites from the original epigenome-wide analyses. FDR: false discovery rate.

**Table 6:** Top gene ontology pathways associated with change in CRP in baseline samples

Gene Ontology term		Overall number of genes in pathway	Number of input genes in pathway	*P*-value	FDR adjusted
GO: 0007156	Homophilic cell adhesion via plasma membrane adhesion molecules	145	17	2.08 × 10^−6^	0.041888
GO: 0098742	Cell–cell adhesion via plasma membrane adhesion molecules	204	17	6.52 × 10^−5^	0.654594

The overall number of genes relates to the total number of genes in the pathway as annotated by Gene Ontology. The number of input genes in the pathway relates to the genes captured by the CpG sites from the original epigenome-wide analyses. FDR: false discovery rate.

### MeQTL results

Methylation values for the replicated DMPs were investigated for meQTLs using SNP data mapped to within 1 million bases around the replicated CpGs using the R package *matrixEQTL* [21]. There was no evidence of meQTLs at any of the replicated loci in this analysis.

## Discussion

In this large EWAS of MTX response in RA, four DMPs that predicted an improvement of SJC and CRP at 6 months were identified in samples taken as early as 4 weeks from RA patients commencing MTX for the first time who were either extremely good or extremely poor responders by 6 months. These genetic loci are novel in the context of RA and MTX response. Bioinformatics evidence suggests that DNA methylation at these loci may modulate cellular adhesion pathways. These biomarkers of response by 6 months were detectable as early as 4 weeks following therapy initiation, potentially representing an advance on current practice by providing an early indication of likely future clinical response and allowing an escalation or change in therapy to optimize efficacy.

In the discovery analysis, no significant changes in methylation were seen between good and poor responders at baseline; however, two DMPs were identified at 4 weeks. This would imply that changes in methylation were due to the effect of MTX acting differently on patients who by 6 months were in extreme responder groups. When analysing the changes in DAS28 components with changes in methylation, there was one CpG, cg04334751, for which changes in methylation correlated with levels of CRP at baseline. This was also replicated by pyrosequencing.

None of the four CpG sites mapped within CpG islands, nor have these regions been associated with MTX metabolism; however, these CpGs map close to genes that are of biological interest in RA. For example, the cg27427581 DMP maps close to the *GATA3* gene region. *GATA3* regulates specific CD4^+^ T helper cell (Th2) differentiation and promotes the expression of Th2 cytokine genes [22]. The cg04334751 DMP maps close to the microRNA gene *MIR-182*. There is emerging evidence that this microRNA is regulated by a number of different transcription factors that promote osteoclastogenesis, which ultimately leads to osteolysis found in RA [23]. *MIR-182* is involved in the TNF-induced osteoclastogenesis programme, which is regulated by transcription factors RBP-J, Foxo3 and Maml1 [23]. This makes the RBP-J–miR-182–Foxo3–Maml1 pathway an attractive candidate for therapeutic targeting [23], and MTX may exert some of its actions via this pathway. Not only does cg04334751 map to a region of potential biological interest, it is also of clinical interest being the only biomarker identified at the pre-treatment time point and was associated with change in CRP. Alone, this DMP is not strong enough to be a single biomarker of treatment response at 6 months; however, it may contribute to a panel of biomarkers in the future that could be used to predict response. Furthermore, while these loci are close to regions relevant to RA pathogenesis, we cannot conclude that these CpG biomarkers are specific to MTX response in RA. To ascertain this, further investigation is required in patients who have other diseases for which MTX is prescribed in low doses such as psoriasis or systemic lupus erythematosus [24, 25].

While interesting CpG associations with improvement of disease activity were identified in this study, integration with genetic data did not find meQTLs at these loci. Finding a more easily typed and stable biomarker such as a SNP, which correlates with the DMP of interest, would be more suitable for clinical use. However, these findings in methylation data should be valued for further exploration as a potential biomarker of response, particularly as DNA methylation is still a more stable measurement when compared with other biomarkers such as transcriptomic signatures.

Strengths of the study include the selection of patients for the first phase genome-wide EWAS; the power of the study was optimized by analysing patients with extreme response status, i.e. from the 2000 patients recruited to the RAMS study, we selected patients who improved most in DAS28 score by 6 months (good responders) and those whose DAS28 improved least or indeed worsened over 6 months. By taking into account the suggestions by Tsai and Bell (2015), our study had over 80% power to identify differences of 7% in methylation [17]. In addition, we adopted a quantitative approach in which changes in methylation were compared with changes in the individual components of the DAS28 at 6 months, in which replication of methylation changes was found to correlate with changes in the more objective components of the DAS28: CRP and SJC. A further strength is that the RAMS cohort includes a representative sample of patients commencing MTX in the UK. Furthermore, samples were available at both the pre-treatment time point and following 4 weeks of therapy, allowing early response biomarkers to be identified. It may be questioned whether the poor responders in a given study are truly poor responders or whether in fact they are non-adherent to the drug [4]. A further strength of the current study is that adherence rate from self-report diaries within the RAMS cohort has been found to exceed 80% at 6 months [26].

Nonetheless, some limitations should also be recognized. It should be noted that RA is a relapsing remitting disease and some changes in the DAS28 over the 6 months may have arisen due to natural variation rather than as a result of the MTX intervention as has been reported for TNFi drugs recently [15]. The study design does not allow separation of prognostic from theranostic biomarkers as no control group was included, but in terms of selecting treatments that will be effective, that may not necessarily matter in the clinical setting [27]. However, this would have made it less likely to see a difference between good and poor responders; therefore, our reported differences in these responder groups are encouraging.

While the study was adequately powered to detect modest changes in methylation status, smaller differences may have been missed. While small differences are less likely to be clinically useful individually, they may be more numerous and provide important added information in an algorithm of response and may also provide mechanistic insight into how MTX exerts its clinical benefit. Therefore, the DMPs alone may not be sufficiently predictive of treatment response to MTX to be clinically useful, especially since the effects were modest; however, the effect was replicated, the patients are real-world and no effect at the same positions was reported in the previous TNFi EWAS study [8]. To conduct an investigation that can be more easily translatable to clinic we chose to test our hypotheses in whole blood samples from RA patients. Since whole blood is a heterogeneous population of blood cells, each with its own distinct methylome, it is important to take into account these differences in analyses. We used the Houseman method [14] to estimate cellular composition based on a reference dataset of the distribution of methylation in these cell types and found no significant differences between the good and poor responders in our study; however, we may have missed more informative DNA methylation biomarkers present in specific cell subsets.

In conclusion, we have found and replicated associations of four DMPs that are identified by 4 weeks of MTX initiation, which correlate with clinical response at 6 months. The findings in the current study are promising and appear to identify methylation patterns that are predictive of improvement of SJC and CRP, which are the more objective components of the DAS28 disease activity score and correlate better with ultrasound synovitis scores. While they are insufficiently predictive alone to be clinically useful, they could be incorporated into algorithms including clinical data and other genetic, epigenetic, genomic and proteomic biomarkers, to provide a more robust predictor of response in RA and use of precision medicine approaches to guide treatment decisions.
